# Unsupervised Human Activity Recognition Using the Clustering Approach: A Review

**DOI:** 10.3390/s20092702

**Published:** 2020-05-09

**Authors:** Paola Ariza Colpas, Enrico Vicario, Emiro De-La-Hoz-Franco, Marlon Pineres-Melo, Ana Oviedo-Carrascal, Fulvio Patara

**Affiliations:** 1 Department of Computer Science and Electronics, Universidad de la Costa CUC, Barranquilla 080002, Colombia; edelahoz@cuc.edu.co; 2 Department of Information Engineering, University of Florence, 50139 Firenze, Italy; enrico.vicario@unifi.it; 3 Department of Systems Engineering, Universidad del Norte, Barranquilla 081001, Colombia; pineresm@uninorte.edu.co; 4 Faculty of Engineering in Information and Communication Technologies, Universidad Pontificia Bolivariana, Medellín 050031, Colombia; ana.oviedo@upb.edu.co

**Keywords:** ambient assisted living—AAL, human activity recognition—HAR, activities of daily living—ADL, activity recognition systems—ARS, clustering, unsupervised activity recognition

## Abstract

Currently, many applications have emerged from the implementation of software development and hardware use, known as the Internet of things. One of the most important application areas of this type of technology is in health care. Various applications arise daily in order to improve the quality of life and to promote an improvement in the treatments of patients at home that suffer from different pathologies. That is why there has emerged a line of work of great interest, focused on the study and analysis of daily life activities, on the use of different data analysis techniques to identify and to help manage this type of patient. This article shows the result of the systematic review of the literature on the use of the Clustering method, which is one of the most used techniques in the analysis of unsupervised data applied to activities of daily living, as well as the description of variables of high importance as a year of publication, type of article, most used algorithms, types of dataset used, and metrics implemented. These data will allow the reader to locate the recent results of the application of this technique to a particular area of knowledge.

## 1. Introduction

### 1.1. Focus of this Survey

Clustering, also called grouping, aims to divide the data into groups of objects with similar characteristics. Through this technique, simplification of data information is achieved. This technique can be applied in both supervised and unsupervised learning, being currently used in different contexts, such as: data recovery, spatial data analysis, marketing, support in medical diagnosis, computational biology, among others. In the specific case of this review of the literature, the results obtained from the application of Clustering to Human Activity Recognition will be analyzed.

- Phase 1. Extraction or Selection of Feature: In this phase, it is necessary to define the characteristics or similarities to be analyzed. This feature can be selected or extracted; the difference in the processes is that, when the selected, the feature is chosen [1], whereas in the second option, the feature is transformed by different techniques to prepare it for new characteristic extracts [2]. The main purpose of this phase is to find the patterns belonging to different clusters, without noise, which are easy to analyze and are known [3,4].
- Phase 2. Clustering Algorithm selection: After extracting the feature, it is necessary to define the clustering algorithm to be applied. In addition to this important selection, defining a corresponding proximity measure and the construction of a criterion function is also indispensable. When the proximity measure function was built, it became an optimization problem with several case studies in the literature [5]. The clustering approach is now applicable to different areas, and for this reason, is very important in order to understand the characteristic of the problem to correctly decide correctly algorithm for solving the identified problem.
- Phase 3. Cluster Validation: In a group of data, the algorithms selected show the different partitions. The big difficulty is to understand and know the quality of the results—the results are defined by the clustering quality metrics [6]. These metrics are divided into two groups: externals and internal. The more useful internal metrics are: cohesion and separation [7], SSW (Sum of Squared Within) [8], SSB (Sum of Squared Between) [7], Sum of Squared base Indexes [6], Davies Bouldin [9], Silhouette coefficient [10] and Dunn-index [11]. The more useful external metrics are: Precision [12], Recall [13], F-Measure [14], Entropy [15], Purity [16], Mutual Information [17,18], and Rand-Index [19]
- Phase 4: Result Interpretation: The purpose of using clustering is to show new information extracted from the original data to solve the initial problem. In some occasions, in order to understand the results, it is necessary to contact an expert in order to explain the cluster’s resultant characteristics. Additionally, additional experiments can be applied in order to explain and prove the extracted knowledge.

### 1.2. The Big Picture: Human Activity Recognition Using Learning Techniques Approach

With the increasing popularity and power of mobile technologies, there is an increasing interest in activity recognition from data collected from sensors in mobile devices [20,21]. One of the challenges of mobile-based activity recognition is the limited computational resource available on mobile devices. Furthermore, activity recognition within smart environments is presented with a number of general challenges. Individuals often carry out activities of daily living (ADL) differently, utilizing a high degree of freedom in relation to the sequential order and duration in which activities are performed. Additionally, a range of sensors within a smart environment typically generates heterogeneous data in terms of both formats and semantics. Consequently, it is often necessary to fuse and interpret sensor data from multiple sources in order to establish the context of an on-going ADL.

Data-driven approaches [22,23] learn activity models from pre-existent large-scale datasets of users’ behaviors using data mining and machine learning techniques. Such techniques are advantageous as they are capable of handling uncertain and temporal information; however, they suffer from the problems of data scarcity, scalability and reusability. By contrast, knowledge-driven approaches build activity models [24,25,26,27] by exploiting rich prior knowledge in the domain of interest to construct models directly using knowledge engineering and management technologies. 

While such models have the advantages of being semantically clear, logical and easy to initialize, they suffer from an inability to handle uncertainty and temporal information. Despite the enormous efforts applied to activity recognition, at present, there exists a gap between the potential of data generation and the aspiration of advanced assistance provision in which context-aware, personalized ADL assistance can be provided whenever needed. Taking into account the reasons explained above, the analysis of the activities of daily life is an area of knowledge rich in challenges where various research groups converge around and have generated, through their experience, different datasets for the experimentation of various learning techniques that are automatic for the analysis and extraction of knowledge. As a result of the different experiments in this line of work, a series of datasets have been built, which will be explained in more detail in the following sections, of which the following stand out: Kasteren [28], CASAS (Kyoto [29], Aruba [30], Multiresident [31]), UCI Human Activity Recognition (HAR) [32], Opportunity [33], and mHealth [34].

### 1.3. Outline

This article is a review of the literature concerning the use of the Clustering technique to discover information in Human Activity Recognition Unsupervised Datasets. First, a taxonomy of the Clustering approach to analyze Human Activity recognition is defined (Section 2). Second, the conceptual information is shown (Section 3). Third, the type of clustering methods for human activity recognition are explained (Section 4). Fourth, the methodology used for the selection and analysis of the results is detailed (Section 5). Fifth, the results of the scientometric analysis and technical analysis are specified, based on the articles selected for the systematic review of the literature (Section 6 and Section 7). Finally, the conclusions and future works are shown (Section 8 and Section 9).

## 2. Taxonomy

**Figure 1:**
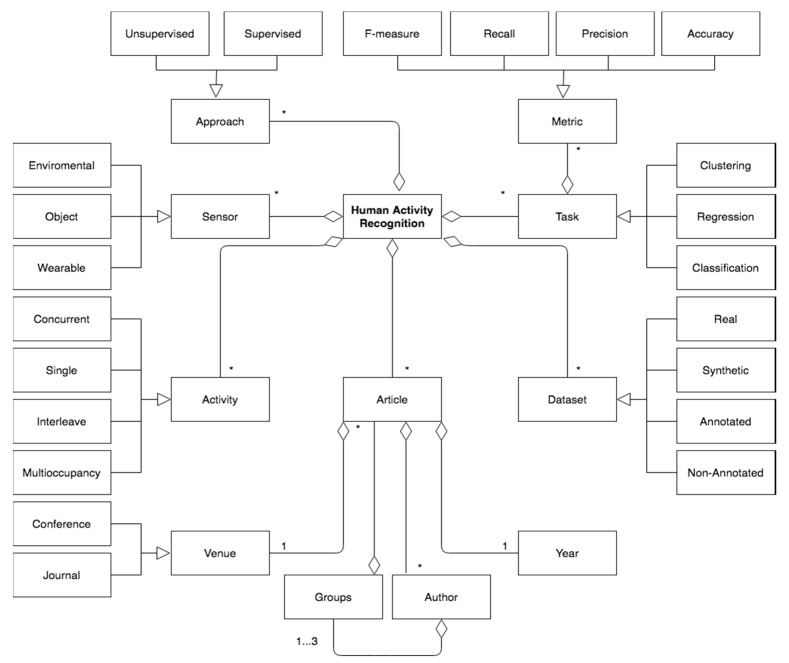
Conceptual model using UML Class Diagram formalism to represent the review of literature concepts.

For the development of the systematic search in the literature, a conceptual or domain model was defined as the fundamental basis to understand the most relevant aspects to be analyzed within the research analyzed, which can be seen in detail in Figure 1. Below is the description and relationships of each of the concepts explained in the diagram.

Human Activity Recognition (HAR) (see Section 3.2) are socialized through Articles that are made at one Venue, whose proceedings can be Conferences or in academic Journals. An Article is published in a specific Year and can be written by many Authors who may belong to different research Groups. To perform the analysis of Human Activity Recognition (HAR), many Datasets are structured. The Dataset can be generated through the diffracted interaction of the users in the houses, which are Real or can be generated in Synthetic form through specialized software. The Dataset can be Annotated when the inhabitants of the houses report, through logbooks or through devices, the activities they perform, or Non-Annotated when this information is not available (see Section 3.2.3).

To analyze Human Activity Recognition (HAR), different tasks of machine learning are used. There are different types of machine learning Tasks that can be used to extract information from these Datasets, of which we can highlight: Classification, Regression, and Clustering. A Classification task consists of being given a fact and is able to identify which predefined class it belongs. Clustering tasks allow you to group objects according to their similarity (see Section 3.1). Regression tasks are used when what you want to find out is a numerical value of a continuous variable. Additionally, the results of the analysis of the quality metrics associated with the implementation of the techniques, both internal and external, can be shown in an Article. 

Human Activity Recognition (HAR), can be analyzed by different Approaches taken in both Supervised and Unsupervised scenarios. A scenario is Supervised when the class criteria information is available. On the contrary, it is Unsupervised when it is devoid of this class criteria information. Human Activity Recognition (HAR) data, is collected through the interaction of people or pets with different types of Sensors, such as: Environmental, Object, and Wearable (see Section 3.2.2). A Sensor is Environmental when it allows for the capturing of different changes in the house, such as: temperature, luminosity, etc. A Sensor is an Object when it is placed on the objects with which people have interactions, such as refrigerators, refrigerators, beds, sofa, etc. Finally, a Sensor is Wearable when it is located directly in the body of the person in order to be able to identify what action it takes, for example, to place sensors in the neck, arms, legs, waist, etc.

The Activities that are captured through the Sensors can be: Single, Interleaves and Multioccupancy. An Activity is Single when in the house of a single inhabitant who performs various activities in a given time; these activities have a special characteristic, i.e., to be able to perform an activity that the previous activity must have culminated. An Activity is Interleave when it is captured through the interaction of a single individual in the house; the main characteristic of this type of activity is that the individual can develop an activity without having completed an activity that has previously started. An Activity is Multioccupancy when it is captured through the interaction of several people in a house that can carry out various activities simultaneously, without having completed the previously initiated activity. Additionally, an Activity is Concurrent when an individual can develop several activities in parallel (see Section 3.2.1).

## 3. Conceptual Information

### 3.1. Clustering Techniques

Clustering is frequently used in any discipline that involves multivariate data analysis. Therefore, there is currently a large amount of literature documenting the importance of grouping together data analysis. Likewise, numerous scientific and application fields that have used clustering techniques, as well as thousands of published algorithms, have also been evidenced. Among the most relevant problems where clustering has been used, we can find: Image Segmentation [27,28,29,30,31,32,33,34,35,36], Document processing [37,38,39], Customer behavior analysis [40,41], Biological analysis of human behavior [42], Grouping and analysis of daily life activities [43,44,45], etc. The use of clustering normally serves the following purposes:

- **Underlying structure**: to depend the data, generate hypotheses, detect anomalies, and identify the most prominent characteristics.
- **Natural classification**: to identify the degree of similarity between the forms of organisms (phylogenetic relationship).
- **Compression**: as a method to organize data and complement it through clustering prototypes.

#### 3.1.1. Clustering Methods

There are currently different methods for the application of clustering. The following will be described: Partitional, Hierarchical, Diffuse, Graphical Based, Evolutionary, Kernel-Based, and Neural Network-Based, see Table 1.

#### 3.1.2. Clustering Methods Descriptions

Partitional Method: The method of partition ordering has, as its main objective, the single partition of data without requiring another additional sub-partition. The result of this type of method is the separation between the groups in the form of hypersurfaces. The main achievement of the partition algorithms is to analyze the distances between the objects that are processed, which have a wide applicability in solving different problems. 

Hierarchical Method: This method aims to optimize a specific function. That is, objects in the same cluster should be like each other, while those in different groups should be as different as possible. The different algorithms framed within this method mainly vary in the measure of similarity and the criteria to evaluate the overall quality of the grouping performed.

Diffuse Method: Clustering algorithms framed in the diffuse method have the purpose of identifying the different classes that represent the functional states that are present in a system, considering the historical dataset that is available. In order to establish the degree of association of the classes that have been identified, it is necessary to have expert staff in the process. By achieving the completeness of these two main tasks, the learning stage of the Diffuse Classifier is completed.

Evolutionary Method: Evolutionary methods are based on employing heuristic targets, which in turn employ other types of computational models for the analysis of the evolutionary process. There are different ways to perform the analysis, based on evolutionary methods, but the following can be highlighted: Genetic algorithms [57,58], Evolutionary Programming Evolutionary Programming [61,67], Evolutionary Strategies [68,69], and Genetic Programming [70].

Spectral Method: Spectral clustering has become one of the most modern ways of performing grouping processes; they are simple to implement and can efficiently solve problems under linear algebraic standards with very good performance, as compared to traditional algorithms, such as K-means [71]. 

Kernel-based method: Spectral clustering methods arise from concepts based on the spectral graph theory. The fundamental idea is to construct a weighted graph for the initial dataset where each of the nodes represent patterns and each weighted edge that changes the values based on each of the patterns. Many of the representations of kernel-based algorithms are based on Laplacian graphs [63,64].

Method based on Neural Networks: Clustering methods, based on neural networks, have become an alternative for solving many real problems [54,55]. The unsupervised neural networks, particularly the SOM self-organizing maps [69,72], provide a more robust and secure use for clustering large amounts of data.

### 3.2. Human Activity Recognition 

Human Activity Recognition (HAR) is the process of automatically detecting human actions from the data collected from different types of sensors [73]. This sensor detects a set of common activities depending of the kind of sensors and the place of occupancy. In fact, this area is an important area of research because it can help solve different problems in sectors, such as: healthcare, energy, computer vision, and others. In this section, the descriptions of the feature of HAR is shown: activities, types of sensors, common datasets, occupancy features and supervised and unsupervised technique examples.

#### 3.2.1. Activities

The main objective of this area of study is to identify the type of activity that is being developed by one or more individuals within a place equipped with a set of sensors that measure different types of parameters. Among the activities that are normally identified are shown in Table 2.

**Figure 2:**
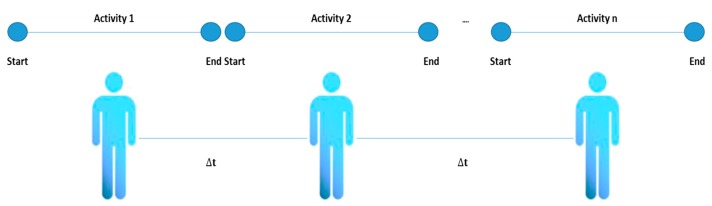
Single activity representation.

**Figure 3:**
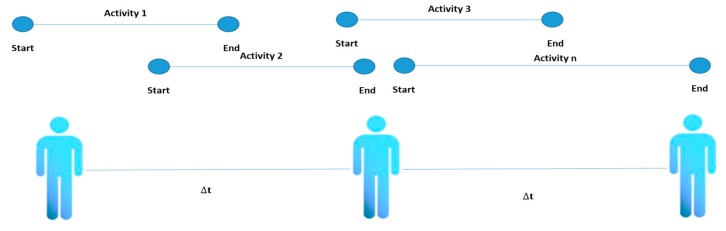
Interleaved activity representation.

**Figure 4:**
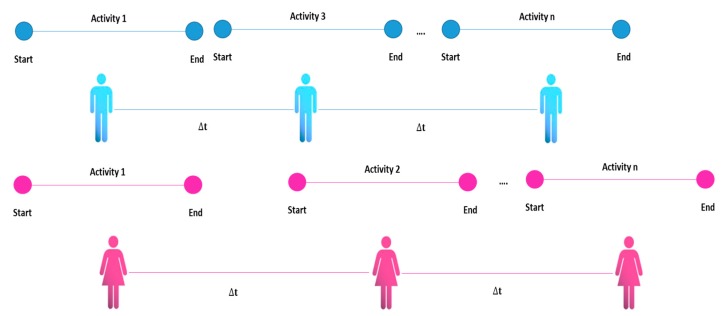
Multioccupancy activity representation.

**Figure 5:**
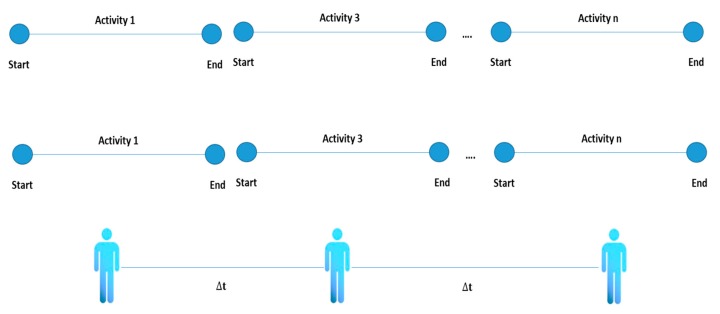
Concurrent activity representation.

Four types of activities have been identified in the literature review: single [32], interleaved [77], multioccupancy [75] and concurrent. The conceptual difference between these types of activities is that a single activity is constituted that is completely terminated before a new activity can be initiated. An interleaved activity refers to one that can be carried out in parallel with another activity and the multioccupancy activities refer to activities carried out by different people simultaneously (see Figure 2, Figure 3, Figure 4 and Figure 5).

#### 3.2.2. Type of Sensor

**Figure 6:**
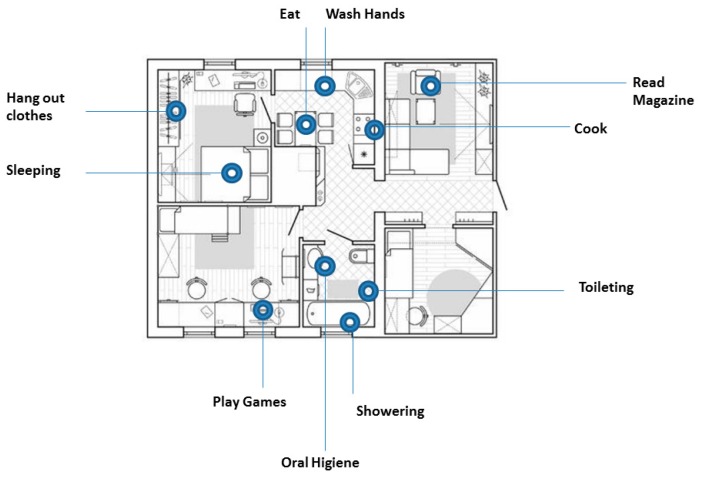
Environmental and object sensor representation related to activities of daily living (ADL).

To complete the process of detecting activities, it is necessary to have sensors of different types. Among which, we can highlight the environmental sensors, object sensors, and wearable sensors. The environmental sensor in an activity recognition context is a passive sensor that are integrated into the environment itself [78,79], such as: robot sensor, combination of audio and video, vision based system, eye tracking sensor, pressure sensors, passive infrared sensor, etc. The object’s sensor shows the state of the objects in an activity recognition context; in some cases, it uses radio frequency identification (RFID). Figure 6 illustrates an example of the activities that can be identified using environmental and object sensors in a common apartment (Table 3) and describes some work related to the use of this type of sensor.

**Figure 7:**
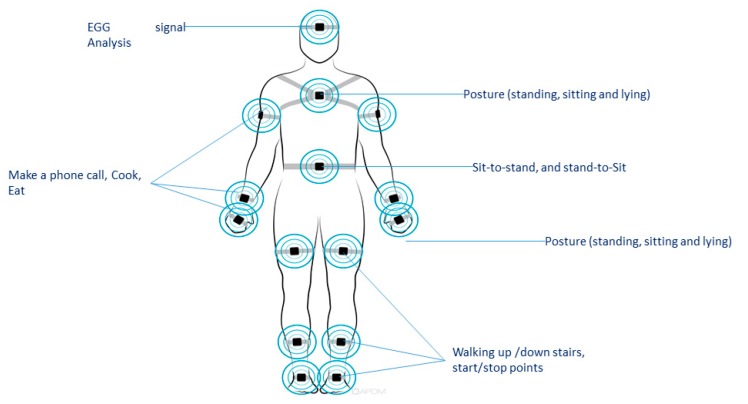
Body sensor representation related to ADL.

The wearable sensors, as the name implies, are integrated into wearable objects or directly with the body in order to help monitor health and/or provide clinically relevant data for care [87]; Table 4 and Figure 7 describe the related work and the use of this type of sensor.

#### 3.2.3. Dataset for Human Activity Recognition

In the literature, many authors developed or used different datasets to identify human activities. In this section, the most useful datasets are explained, taking into account different aspects, such as: occupancy, number of individuals that interact, number of activities, and the type of sensor, with the following acronyms being used. E—Environmental Sensor, O—Object Sensor, A—Accelerometer, G—Gyroscope, and M—Magnetometer (see Table 5).

The Van Kasteren dataset [28] recorded the data of single occupancy in houses with one man. He stays alone in the apartment with three rooms. In this apartment, there were 14 wireless sensors. The data was collected for 28 days; the results of the interaction of the man with the sensors were: 2120 sensors events and 245 instances of activities. The Opportunity dataset [33] shows the interaction of four people in a house. In this house, there were 23 body-worn sensors, 12 object sensors, 21 ambient sensors and 4 recordings. Finally, this dataset contains 242 attributes and 2551 instances of activities. 

The CASAS Daily Life Kyoto [29] is a single occupancy dataset that was collected from 25 October 2007–31 May 2008. In this apartment, there were 40 sensors consisting of: 37 motion and 3 temperature sensors; this dataset contains 4 attributes and 1530 instances of activities. The UCI Human Activity Recognition Using Smartphones DatasSet [32] details the interaction of 30 subjects, within an age bracket of 19–48 years. The subjects perform 6 activities: walking, walking upstairs, walking downstairs, sitting, standing and laying. This dataset contains 561 attributes and 10,299 instances.

The CASAS Aruba [30] is a single occupancy dataset; the volunteer woman interacts with 4 temperature sensors, 31 motion sensors, and 4 door closure sensors. This dataset contains 5 attributes, and 6438 activities were identified. The PAMAP2 dataset [95] shows the interaction of 9 people for approximately 1 month. The records detail 19 physical activities, such as: standing, walking, ascending stairs, and descending stairs. 

The CASAS Kyoto Multiresident [31] shows the interaction of 2 individuals in an apartment at the same time, with 78 sensors: 51 motion, 8 item, 12 cabinets, 2 water, 1 burner, 1 phone, and 3 temperature sensors, distributed in several places. The records show 5 attributes: date Time, SensorID, Value ResidentID, and TaskID. The first three are generated automatically for the smart interactions and the last two are annotated by the residents. The USC-HAD [96] shows the interaction of 14 people (7 male and 7 female), performing 12 activities: walking forward, walking left, walking right, walking upstairs, walking downstairs, running forward, jumping, sitting, standing, sleeping, elevator up, and elevator down. The record shows 14 attributes, which were generate using different types of sensors: accelerometer and a motion binary sensor.

The mHealth dataset [34] shows the interaction of 10 people in an apartment. During the experiment, 3 wearable sensors sent information concerning the activities carried out by the people. These activities consisted of standing still, sitting and relaxing, lying down, walking, climbing stairs, waist bends forward, front elevation of arms, knees bending (crouching), cycling, jogging, running, and jump front and back. The record contains 23 attribute and 120 instances. The WISDM dataset [99] details the activities of 29 people in an apartment performing 6 activities: walking, jogging, sitting, standing, and climbing stairs. The data was collected using mobile phone with the Android operating system.

The MIT PlaceLab [98] is a single occupancy dataset. The data was collected on Friday, 4 March 2005 from 9 a.m. to 1 p.m., performing 10 activities. In the apartment, there was a variety of sensor as switch sensors, light sensors, and current sensors, etc. The data were labeled by the people who participated in the experimentation. The Daily and Sports Activities Dataset (DADS) [99] shows the interaction of 8 people (4 males and 4 females between the ages 20 and 30) for 5 min. The persons performing 19 activities: sitting, standing, lying on back and on right side, ascending and descending stairs, standing in an elevator still, moving around in an elevator, walking in a parking lot, walking on a treadmill with a speed of 4 Km/h, walking on a treadmill with a speed of 8 Km/h, exercising on a stepper, exercising on a cross trainer, cycling on an exercise bike, rowing, jumping, and playing basketball. For recovery of the data, 9 sensors were used: x,y,z accelerometers, x,y,z gyroscopes, and x,y,z magnetometers. This dataset contains 5625 attributes and 9120 instances.

Finally, three datasets are described in the DOMUS dataset [100]; the data was collected in an apartment with 40 square meters in order to detect 15 activities. This apartment was provided by a variety of sensors types: electricity counter, water counters, ceiling spots, presence detector, power plugs, dimmed plug, temperature sensor, external shutters, internal blinds, luminosity sensor, air quality sensor, controllable curtains, dimmed light, and luminosity sensor. The Smart Environment-Ulster University [101] dataset detects the following activities: drink a glass of water, prepare tea, prepare a hot chocolate, drink a glass milk, call by phone, prepare hot snack, prepare cold snack, watch TV, and washing dishes. To detect these types of activities, different types of sensor were used: kitchen door sensor, living room door sensor, cutlery cupboard sensor, dishes cupboard sensor, glasses and cup cupboard sensor, pantry cupboard sensor, fridge door sensor, chair sensor, sofa sensor, television sensor, phone sensor, water sensor, and kettle sensor. The UJAmI SmartLab [102], by Universidad of Jaén in Spain, shows the interaction of different types of sensors: environmental wearable sensors; actuators; smart device; low cost devices, like raspberry Pi and arduino; indoor locations; vision cameras; screen; health devices; brain interfaces; human computer interfaces; and robots. 

#### 3.2.4. Supervised and Unsupervised

In the construction of the data, it is important to specify that it is possible to perform different types of analyses, taking into account whether or not you have the class criteria that defines the activity that the individual is doing inside the house. In the case of having information on the kind of activity that is being developed, either one or several individuals or even pets, it is determined that the learning that can be done on the data is supervised, or also in terms of the area of knowledge annotated. These types of annotations are usually made through the completion of activity logbooks, or alternatively, using different devices, either audio or video. When there is no information on the kind of activity that is being carried out by either an individual or several individuals in the home, the learning that can be done on this data can be called unsupervised, or it can also be called non-annotated. In other occasions, the same dataset allows the two types of both supervised and unsupervised learning to be carried out, so it is partially annotated.

#### 3.2.5. Single or Multioccupancy 

A highly important factor in the field of recognition of daily life activities is the definition of the number of people or animals that interact within the house. This variable is really very important because the habits of the activities in each of the individuals vary. For example, in some datasets, the participation of the experimentation of older adults and children is identified. Additionally, interaction with pets that can activate the action of a sensor inside the house for a certain time without performing any activity can be included in the data. All aforementioned variables complicate the model and therefore generate new research topics in this area of knowledge.

## 4. Type of Clustering Methods for Human Activity Recognition

Taking into account the fact that clustering is one of the techniques mostly used in issues of extraction and the selection of similarities and common characteristics between objects, different architectures or forms of implementation have been developed that allow its correct application in the recognition of activities of daily life. The similarities of the implementation of different clustering techniques allow its view to become the basis for the subsequent implementation of other techniques or tasks of machine learning, thus showing the versatility of applications that clustering can have in this context. These architectures or forms of knowledge extraction are shown in the extensions below:

With the generation of new datasets, the analysis of the data quality and the beginnings of implementation arises. Many of the datasets generated can be analyzed through either the execution of a technique or by comparing several existing techniques to later analyze the results of the quality metrics associated with the implementation. Other more mature experiments focus on the selection of a different HAR dataset, which contain similar characteristics associated with: occupancy, type of sensors, among others, in order to identify the results of different techniques when being implemented in the selected dataset.

## 5. Methodology

**Figure 8:**
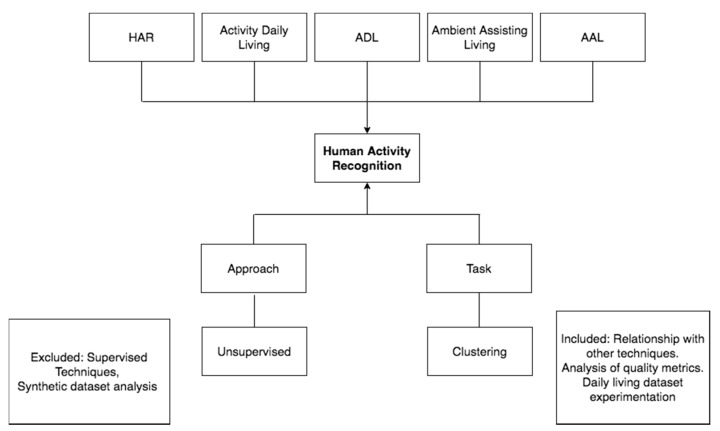
Search features of review. HAR—Human Activity Recognition; ADL—Activities of Daily Living; AAL—Ambient Assisting Living.

To carry out this review of the literature effectively, different tools and supplies were considered. The searches were carried out in the specialized databases: Scopus, Web of Science, Science Direct, IEEE, and ACM. The methodology for the presentation and analysis of the results is referenced by Kitchenham [103]. Figure 8 illustrates the different concepts that were considered to perform the search.

It is important to specify that the area of study in question is limited to the analysis of the different activities that you can perform, either an individual or several, in a smart place. That is why different acronyms or keywords have been defined in the literature to identify this area of study. Among which, the following can be identified: Human Activity Recognition (HAR), Activity Daily Living (ADL), and Ambient Assisting Living (AAL). Additionally, only articles published between 2015 and 2020 were selected. Other very relevant aspects are the identification of the approach that can be supervised or unsupervised for the specific case of this review, the work carried out on human activity recognition with a “supervised” approach, and the “images and audio” feature was excluded. In the same way, there are different tasks of data mining that have been used to recognize the activities of daily life; punctually in this review refers to those that, in any of their phases of knowledge discovery, have made use of “Clustering” to support the interpretation or improvement of the results.

With the combination of keywords mentioned above, a total of 190 records were obtained, which were subsequently filtered through the relevance of the appearance of the keywords in the document, the application of the technique in the discovery of knowledge and the contribution of the combination of clustering with other techniques to solve problems associated with this study area, and finally obtaining a set of 64 records that will be analyzed in later sections of this document. These records were analyzed using a meta-analytical matrix that has the following fields: year of publication, title of the paper, name of the journal or event where the article was published, ISSN, venue type in which the article was published as either a journal or conference, quartile if applicable, country where the journal originated from or where the conference was held, country of the first author, university to which the first author is a part, dataset used for experimentation, type of dataset whether synthetic or real, type of task performed, algorithm used, and metrics extracted from the application of the selected algorithm. With these aspects, it is possible to detail the results of the various experiments carried out.

In this matrix, general information of the publications are analyzed (see Table 6). Using the information of general publications, different conclusions of high relevance can be made, such as: the year of the greatest number of publications on the specific subject, which can determine the trend line in the publications, and the database where the majority of the authors selected to make the publications related to the topic. The titles that are constrained within the selected keywords to perform the search can also be identified. In the journal column, the journal, conference, or book series where the results regarding this line of work are usually published can be identified.

In the same way, by using this matrix, the quartile of the publications, as well as the country where the subject is being published and the university and country of the first author, can be identified. All this information supports the identification of the research groups or researchers that are supporting the advances in the development of this line of knowledge.

Among the most relevant technical aspects of the meta-analytical matrix is the identification of the clustering algorithm and the different analyses of the quality of the techniques associated with the different experiments (see Table 7).

## 6. Scientometric Analysis 

**Figure 9:**
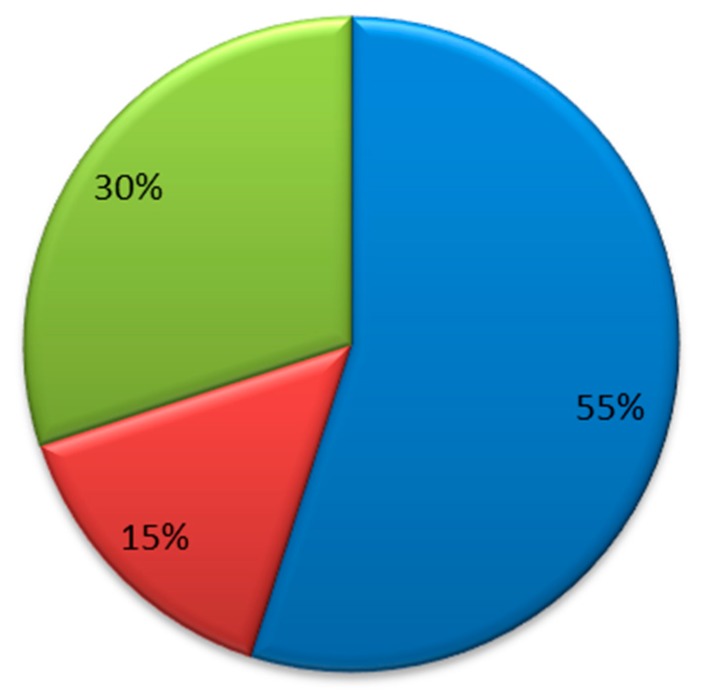
Origin of the publications by venue.

Considering the analysis of the 64 records, which were processed based on the identification of the use of the clustering technique in the recognition of unsupervised daily life activities, the following scientific data could be identified, as detailed below. In Figure 9, it can be noted that 55% of the publications have been made in the Journal, while 30% of the publications are from participation in conferences where those that have been organized by IEEE stand out. Only 10% correspond to the Book Series.

**Figure 10:**
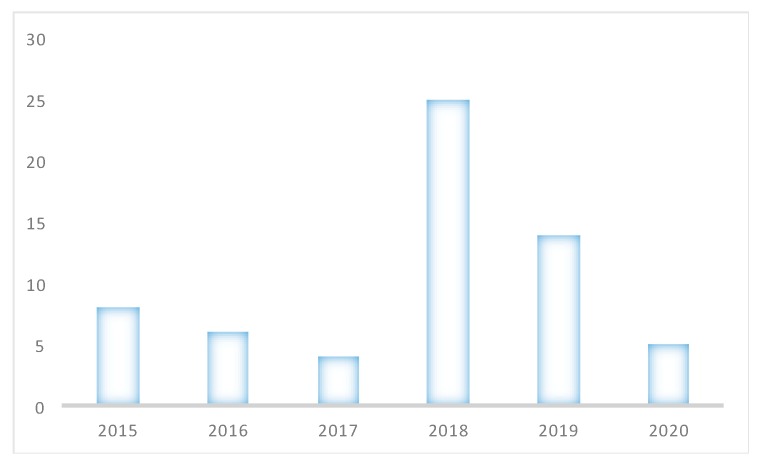
Analysis of the articles according to the year of publication.

Regarding the years of publication of the manuscripts, it is important to specify that the search was confined to between 2015 and 2020. The year 2018 had the highest concentration of publications, and the year 2017 was consolidated as the year where the lowest number of publications was found, as can be seen in Figure 10. 

**Figure 11:**
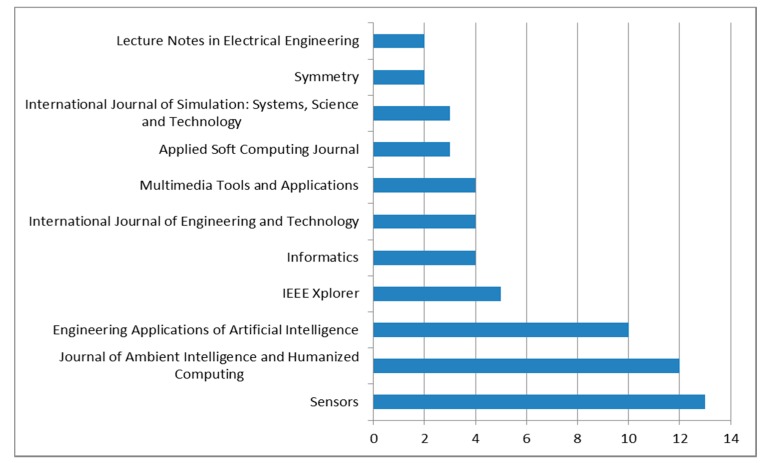
Distribution of articles according to the journal in which they were published.

In the same way, when performing an analysis of the articles published only in journals, we excluded those published in conferences and book series. The Sensors journal is consolidated as the journal with the highest number of works in this work area being published, based on the articles of this review, followed by the journal of Ambient Intelligence and Humanized Computing and the Engineering Applications of Artificial Intelligence (see Figure 11).

**Figure 12:**
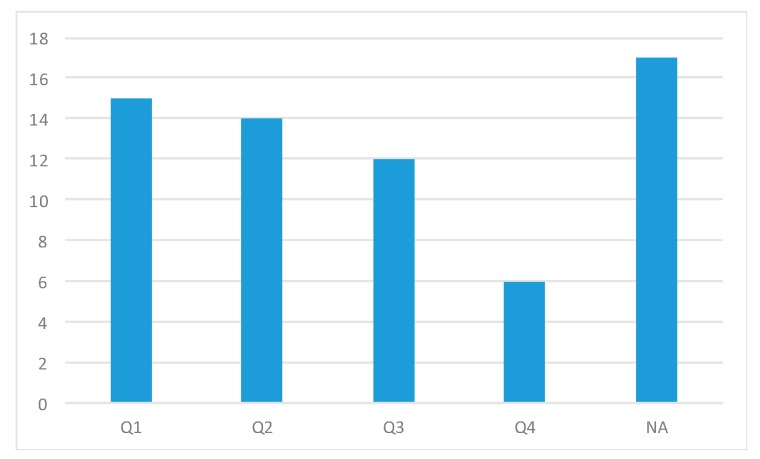
Quartile of journal publications.

Figure 12 shows the quartiles in which the set of publications is delimited, excluding those belonging to conference proceedings. The publications that have been taken as the basis for this review are in quartile Q1, followed, respectively, by quartile Q2, having, as a minority, those published in journals Q4.

**Figure 13:**
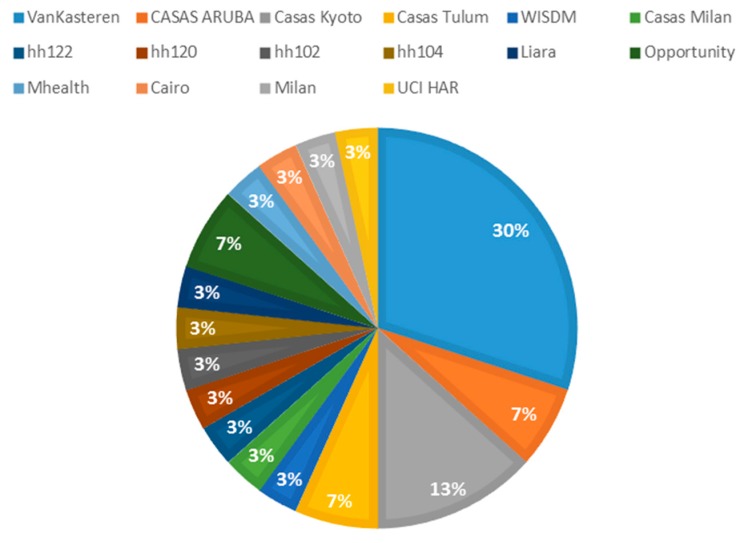
Search features of the review.

When carrying out an analysis of the most used dataset for experimentation with clustering in an unsupervised environment, the wide use of the VanKasteren dataset with 30% can be highlighted, followed by the Casas Kyoto dataset with 13%. The Casas Aruba and Opportunity dataset have also been used in 7% of experiments (see Figure 13).

## 7. Technical Analysis

There is a wide range of clustering techniques within the literary review, which have been used to perform various experiments in different contexts. In this review, we will concentrate on three techniques that have been most used to analyze the recognition of activities of daily living: K-NN, K-means, and Sub-clustering. While K-means and sub-clustering are concerned with finding similarities between the data and forming groups, the K-NN technique is used to predict new data to which cluster they belong. The combination of K-means to find groups and K-NN to predict new instances is usual because both algorithms are based on distance measurements. In Table 8, Table 9 and Table 10, each of the respective techniques have been discriminated, considering the high relevance of variables, such as the reference to the author who performed the experimentation, the dataset used, and the quality metrics associated with the experimentation. For the specific case of this analysis, the metrics of quality accuracy, precision, recall and F-measure have been used, the data being entered in accordance with the data provided by the authors.

One of the mostly used algorithms is the K-NN algorithm. The experiments of this algorithm have been carried out with the dataset widely detailed in the literature, such as: VanKasteren, Casas Aruba, Casas Kyoto, Casas Tulum, Hh102, Hh1034, UCI HAR, MIT PlaceLab, and PAMAP2. In Reference [104], the best results of the K-NN implementation in the VanKasteren dataset can be seen, obtaining results, such as: 97.2% accuracy, 88.25% accuracy, 83.6% recall, and 84% F-measure. In the same way [104], the results of the implementation of this technique in the Casas Aruba and Casas Kyoto dataset are detailed, with the following quality metrics: Accuracy 98.14%, precision 74.73%, Recall 76.29%, and F-measure 72%. Another experimentation carried out in References [104] in the Casas Tulum dataset, shows the following results: Accuracy 86.15%, Accuracy 59.18%, Recall 57.12%, and F-measure 57%. Similarly, the experiments carried out in the following datasets stand out, which show the following results of the metric accuracy: Casas Tulum [104] 86.15%, Hh102 [105] 66%, Hh104 [105] 78%, UCI HAR [106] 71%, MIT PlaceLab [107] 94.5%, and PAMAP2 [108] 62%. Table 7 shows the result of the implementation of the K-means algorithm in different datasets, in which the following accuracy results can be highlighted: VanKasteren [109] 88.6%, WISDM [110] 71%, Liara [111] 86%, MHealth [112] 71.66%, Opportunity [113] 86.8%, and UCI HAR [114] 52.1%.

In Reference [115], the author showed the experimentation of the sub-clustering technique at different datasets, obtaining the following accuracy results: VanKasteren 94.3%, Casas Aruba 91.88%, Casas Kyoto 96.67%, Casas Tulum 99.28%, Milan 95.20%, and Cairo 94.17.

## 8. Conclusions

The objective of this article is to show to the community of researchers of recognition of human activities, or HAR, some recommendations about the different Clustering techniques used for the analysis of different types of dataset in an unsupervised experiment. It is important to point out that this review is framed in articles that were published between 2015 and 2020. The database that is currently publishing the largest number of articles focused on clustering applications to HAR is IEEE Xplorer, with 66% of the publications when comparing results with other databases, such as Scopus, Science Direct, Web of Science, and ACM.

Additionally, it is important to point out that the vast majority of the works, 70%, come from conference articles, while the rest of the articles have been published in journals or Book Series. The USA is the country that has accepted the largest number of publications in this area of knowledge, followed by Germany and the Netherlands. One of the most published journals in this line of knowledge is Sensors and the Journal of Ambient Intelligence and Humanized Computing. Among the institutions that stand out the most for the development of this theme are: WSU (USA) and the University of Ulster (Ireland).

Considering the datasets that have been used to carry out the different unsupervised experiments, VanKasteren has been the dataset most used by the authors (30%), followed by Washington State University with its CASAS project, where the datasets: Kyoto (13%), Tulum (7%), and Aruba (3%) are the ones that have been used by the authors.

Within the experiments analyzed, it is possible to identify the effectiveness of the clustering techniques or algorithms in unsupervised environments, as detailed in Table 11. Of all the datasets implemented, Casas Aruba, VanKasteren, and Casas Tulum have been the ones that have shown the best results in the unsupervised clustering implementation processes, with the following accuracies, 98.14%, 88.6%, and 99.28%, respectively.

The analysis described above allows for the analyzing of the wide usability of partition algorithms to analyze the dataset of activities of daily life, as well as their effectiveness in the evaluation of the quality metrics of the results of the experiments.

## 9. Future Works

**Figure 14:**
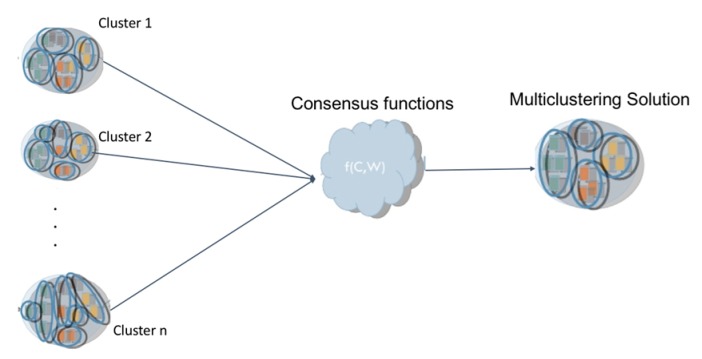
Multiclustering application architecture.

Among the future works planned, after this systematic review of the literature, is to explore other types of techniques that have not been used as methods, based on hierarchies or kernel for the identification of this type of activity; likewise, is the objective to perform different experiments with different methods that support, with greater precision, the identification of the activities of daily life. Other aspects or challenges of this line or area of research can be highlighted:

- Usability of clustering techniques in conjunction with other techniques or algorithms, such as HMM, which support the unsupervised detection of daily life activities.
- Generation and use of new techniques to analyze temporal space support to improve the results of the identification of activities of daily life.
- Other challenges within the clustering application can identify the behavioral analysis of each of the groups generated. This analysis is called Multiclustering Methods, which creates multiple groupings and then combines them into a single result (see Figure 14).
- Exploration of different experimentation scenarios with multi-level applications that include the behavior of unidentified activities.

## Figures and Tables

**Table 1:** Clustering’s methods and algorithms.

Method	Algorithm
Partitional Method	K-means algorithm [46,47]
Hierarchical Method [48]	COBWEB [49,50]
Diffuse Method [13,51]	Fuzzy C Means [52,53]
Method Based on Neural Networks [54,55]	SOM [56]
Evolutionary Methods [57,58,59]	Genetic Algorithms [57,58,59,60,61,62]
Kernel-Based methods [63,64]	Kernel K-means Algorithms [65,66]
Spectral Methods [36]	Standard Spectral Clustering [59]

**Table 2:** Useful activity in Human Activity Recognition.

#	Activity’ Name	Description
1	Make a phone call [74]	The participant moves to the phone in the dining room, looks up a specific number in the phone book, dials the number, and listens to the message.
2	Wash hands [74]	The participant moves into the kitchen sink and washes his/her hands in the sink, using hand soap and drying their hands with a paper towel.
3	Cook [74]	The participant cooks using a pot.
4	Eat [74]	The participant goes to the dining room and eats the food.
5	Clean [74]	The participant takes all the dishes to the sink and cleans them with water and dish soap in the kitchen.
6	Fill medication dispenser [32]	The participant retrieves a pill dispenser and bottle of pills.
7	Watch DVD [32]	The participant moves to the living room, puts a DVD in the player, and watches a news clip on TV.
8	Water plants [32]	The participant retrieves a watering can from the kitchen supply closet and waters three plants.
9	Answer the phone [32]	The phone rings, and the participant answer it.
10	Prepare birthday card [32]	The participant fills out a birthday card with a check to a friend and addresses the envelope.
11	Prepare soup [32]	The participant moves to the kitchen and prepares a cup of noodle soup in the microwave.
12	Choose outfit [32]	The participant selects an outfit from the clothes closet that their friend will wear for a job interview.
13	Hang up clothes in the hallway closet [75]	The clothes are laid out on the couch in the living room.
14	Move the couch and coffee table to the other side of the living room [75]	Request help from another person in multioccupancy experimentation.
15	Sit on the couch and read a magazine [75]	The participant sits down in the living room and reads a magazine.
16	Sweep the kitchen floor [75]	Sweep the kitchen floor using the broom and dustpan located in the kitchen closet.
17	Play a game [75]	Play a game of checkers for a maximum of five minutes in a multioccupancy context.
18	Simulate paying an electric bill [75]	Retrieve a check, a pen, and an envelope from the cupboard underneath the television in the living room.
19	Walking [76]	Using body sensors, define if the participant is performing the walking action.
20	Sitting [76]	Using body sensors, define if the participant is performing the sitting action.
21	Sleeping [76]	Using body sensors, define if the participant is performing the sleeping action.
22	Using a computer [76]	The participant is in the position of use of the computer for a certain time.
23	Showering [76]	Detection of environmental sensors of the participant’s stay in the shower.
24	Toileting [76]	Detection of environmental sensors of the participant’s stay in the bathroom.
25	Oral hygiene [76]	Using the object and body sensors, the oral hygiene action is identified.
26	Making Coffee [76]	Detection of objects and environmental sensors of the action of making coffee by the participant.
27	Walking upstairs [76]	The participant performs the action of climbing the stairs, being detected by the body sensors.
28	Walking down stairs [76]	The participant performs the action of going down the stairs, being detected by the body sensors.

**Table 3:** Body sensor analysis.

#	Type of Sensor	Sensor	Type of Activities	Reference
1	Environmental and Object sensors	Motion detectors, break-beam, pressure mats, contact switches, water flow, and wireless object movement	Eat, drink, housework, toileting, cooking, using a computer, watching TV, and call by phone	[80]
2		motion, temperature and humidity sensors, contacts switches in the doors, and item sensors on key items	phone call, cooking, wash hands, and clean up.	[81]
3		Binary sensors on doors and objects	Toileting, bathing, and grooming	[82]
4	Object sensors	Shake sensors	Leaving, toileting, showering, sleeping, drinking, and eating	[83]
5		radio frequency identification (RFID)	Toileting, oral hygiene, washing, telephone use, taking medication, etc.	[84]
6			Using bathroom, making meals/drinks, telephone use, set/clean table, eat, and take out trash	[85]
7			Making coffee	[86]

**Table 4:** Body sensor analysis.

Number	Sensor Location	Type of Activities
1	Chest [88]	Standing, sitting and lying.
2	Waist [89]	Sit-to-stand, stand-to-sit, walking.
3	Upper arm, wrist, thigh and ankle [90]	Posture and some ADLs.
4	Wrist [91]	Sport movement.
5	Wrist, waist, and shoulder [92]	Riding elevator, walking up stairs.
6	On the belt [93,94]	Walking upstairs, walking downstairs, start or stop points.

**Table 5:** Dataset’s descriptions.

Number	Dataset’s Name	Occupancy	# Subjects	# Activities	Sensor‘s Type
1	Vankasteren [28]	Single	1	8	E
2	Opportunity [33]	Multioccupancy	4	16	O, A
3	CASAS- Daily Life Kyoto [29]	Single	1	10	O, A
4	UCI SmartPhone [32]	Multioccupancy	30	6	A, G
5	CASAS Aruba [30]	Single	1	11	E, O
6	PAMAP2 [95]	Multioccupancy	9	18	A, G, M
7	CASAS Multiresident [31]	Multioccupancy	2	8	A, O, E
8	USC-HAD [96]	Multioccupancy	14	12	A, G
9	mHeath [34]	Multioccupancy	10	12	A, G
10	WISDM [97]	Multioccupancy	29	6	A
11	MIT PlaceLab [98]	Single	1	10	A, O, G
12	DSADS [99]	Multioccupancy	8	19	A, G, M
13	DOMUS [100]	Single	1	15	A, G, O
14	Smart Environment- Ulster University [101]	Single	1	9	A, G, M
15	UJAmI SmartLab [102]	Single	1	7	O, E

**Table 6:** General information of publication analyzed in a meta-analytical matrix.

Identifier	Year	Paper Title	Journal	ISSN	Proceedings or Book	Quartile	Journal Country	First Author´s Country	University
Art1	2015	Towards unsupervised physical activity recognition using Smartphone accelerometers	Multimedia Tools and Applications	1380–7501	Book Series	Q1	Netherlands	China	Langhou University

**Table 7:** General information of publication analyzed in a meta-analytical matrix.

Identifier	Dataset	Type	Methods	Metrics				Approach
				Accuracy	Precision	Recall	F-Measure	
Art1	Kasteren	Real	Calculating neighborhood radius	86	76	80	76	Unsupervised
Art2	WISDM	Real	MCODE-Based	85	77	83	77	Unsupervised

**Table 8:** Details of the K-NN experimentation.

References	Dataset	Accuracy	Precision	Recall	F-Measure
[104]	Van Kasteren [28]	97.2%	88.25%	83.66%	84%
[116]		96.67%	97.33%	96.67%	97%
[117]		93.55%	92.97%	91.3%	91%
[109]		--	95%	100%	97%
[118]		88.14%	--	--	--
[107]		97%	--	--	--
[119]		92%	--	--	--
[120]		78.9%	--	--	--
[121]		84%	--	--	--
[122]		89.5%	--	--	--
[123]		82%	--	--	--
[104]	Casas Aruba [30]	98.14%	74.73%	76.29%	72%
[123]		77.10%	--	--	--
[124]		74%	--	--	--
[125]		78%	--	--	--
[126]		98.93%	--	--	--
[127]		73.44%	--	--	--
[104]	Casas Kyoto [29]	98.14%	74.73%	76.29%	72%
[116]		94.21%	90.10%	93.11%	91%
[117]		94.62%	93.21%	94.62%	93%
[128]		91%	--	--	--
[129]		89%	--	--	--
[130]		81.1%	--	--	--
[131]		--	83.26%	--	--
[132]		87.45%	86.12%	--	--
[125]		78%	--	--	--
[104]	Casas Tulum [67]	86.15%	59.18%	57.12%	57%
[131]		--	--	--	72%
[132]		--	--	--	74%
[125]		--	65.3%	82%	--
[104]		75.45%	--	78%	--
[105]	Hh102 [68]	66%	--	--	53%
[105]	Hh104 [68]	78%	--	--	60%
[115]	UCI Human Activity Recognition (HAR) [90]	71%	--	--	--
[107]	MIT PlaceLab [97]	94.5%	--	--	--
[118]	PAMAP2 [92]	62%	--	--	--

**Table 9:** Details of the K-means experimentation.

References	Dataset	Accuracy	Precision	Recall	F-Measure
[46]	VanKasteren [28]	--	88.6%	95.48%	91.91%
[133]		87.21%	--	--	--
[134]		82%	--	--	--
[135]		--	--	72%	85%
[136]		--	--	--	82.78%
[137]		--	76.23%	--	--
[138]					
[139]	WISDM [96]	71%	--	--	--
[140]	Liara [93]	86%	--	--	--
[109]	Opportunity [33]	79%	--	--	--
[128]		80%	--	--	--
[115]		86.8%	--	--	--
[138]		--	79.67%	--	--
[141]		--	82.45%	--	--
[142]		--	--	75.45%	--
[143]		--	--	--	87.32%
[144]		--	--	--	85.45%
[139]	MHealth [34]	71.66%	--	--	--
[112]		71%	--	--	--
[145]		78.45%	--	--	--
[146]		--	--	--	78.56%
[147]		--	--	--	77.56%
[148]		73.45%	--	--	--
[149]		78.63%%	--	--	--
[150]	UCI HAR [32]	52.1%	--	--	--
[151]		76.32%	--	--	--
[152]		--	--	--	77.22%
[153]		--	--	--	78.45%
[154]		79.37%	--	--	--
[155]		75.31%	--	--	--

**Table 10:** Details of the sub-clustering experimentation.

References	Dataset	Accuracy
[156]	VanKasteren [28]	94.3%
[157]		78.5%
[158]		75.42%
[159]		81.65%
[160]		86.32%
[161]		89.45%
[156]	Casas Aruba [30]	91.88%
[161]		88.32%
[162]		89.78%
[163]		87.67%
[164]		86.43%
[165]		89.12%
[156]	Casas Kyoto [29]	96.67%
[166]		86.32%
[167]		76.45%
[168]		89.12%
[169]		85.34%
[156]	Casas Tulum [67]	99.28%
[156]	Milan [68]	95.20%
[156]	Cairo [68]	94.17%

**Table 11:** Details of the best results of the experiments.

References	Dataset	Technique	Accuracy
[110]	Casas Aruba [30]	K-NN	98.14%
[119]	VanKasteren [28]	K-means	88.6%
[125]	Casas Tulum [67]	Sub-Clustering	99.28%
